# Caries Experience and Periodontal Status during Pregnancy in a Group of Pregnant Women with HIV+ Infections from Puerto Rico

**DOI:** 10.4172/2155-6113.1000434

**Published:** 2015-02-27

**Authors:** Lydia M López, María Elena Guerra

**Affiliations:** 1 Surgical Sciences Department, University of Puerto Rico School of Dental Medicine, San Juan, Puerto Rico, USA; 2 Pediatric Dentist and Professor, Central University of Venezuela, School of Dentistry, CAPEI (Centro de Atención a Pacientes con Enfermedades Infectocontagiosas) Dental Clinic, Caracas, Venezuela, USA

**Keywords:** HIV+/AIDS, Periodontal disease, Viral load, HIV+ infections

## Abstract

**Objectives:**

The aim of this study was to determine the caries rate and periodontal status in a sample of pregnant women with HIV+ infections from Puerto Rico.

**Methods:**

A pilot study was conducted on a cross sectional convenience sample of 25 pregnant women with HIV+ infections from Puerto Rico who visit the CEMI clinic (Centro de Estudios Materno Infantil) at the University of Puerto Rico. The women subjects were evaluated for caries, DMFT (D: Decay tooth; M: Missing tooth due to caries; F: Filled tooth) index, oral lesions associated with HIV+/AIDS and periodontal disease parameters, with a Florida probe by a calibrated dentist on periodontal indexes such as as bleeding on probing, CEJ (cemento-enamel junction) and pocket depth. Periodontal disease was classified as having 4 sites with pocket depth greater than 4 mm and caries were identified following the Radike criteria. Data was statistically analyzed using the SSPS Program (Statistical Software Program for Social Sciences) and descriptive statistics were calculated.

**Results:**

Mean DT (decayed teeth), MT (missing teeth due to caries), FT (filled teeth) and DMFT (decay, missing and filled teeth) were 4.8, 1.86, 5.3 and 12, respectively; mean sites of bleeding on probing=12.06; mean sites with pocket depth>4 mm=6.95 and mean sites with loss of attachment greater than 4 mm=7.66. [Almost 50% of the patients had generalized chronic periodontitis. A 72% prevalence of periodontal disease was found. No oral lesions related to HIV+/AIDS were reported. CD4 and viral load was statistically associated with bleeding on probing and severe signs of periodontal disease.

**Conclusions:**

High levels of dental disease were found in pregnant women with HIV+/AIDS infections from Puerto Rico, and these women were in need of substantial dental services.

## Introduction

The UNAIDS (UNITED NATIONS AIDS PROGRAM) 2011 Global Report states that as of the end of 2010, 34 million individuals were living with HIV+/AIDS, and this represents an increase of 17% as compared to those living with HIV+/AIDS in 2001. This improved survival is related to greater access to treatment with retroviral medications that have proven to be most effective in reducing the death rate from AIDS.

The proportion of women and girls living with HIV+ has been stable at 59% of the world's total HIV+ population, and 53% of the HIV+ population of the Caribbean region. The Caribbean region has the second highest prevalence of HIV+ preceded by Sub-Saharan Africa. In the Caribbean region, the number of new HIV+ infections has decreased one-third since 2001, in part because pregnant women with HIV+ infections have had improved access to prevention and treatment that has decreased the number of children born with HIV+. In general, the HIV+ epidemic in the Latin American region continues to be stable and the number of individuals living with HIV+ has increased in recent years [1].

Periodontal disease is a chronic inflammatory process involving specific bacteria that affect the tissue and bone supporting the teeth. While periodontal disease can occur in anyone regardless of HIV+ status, one particularly severe form of the disease (necrotizing ulcerative periodontitis) and a related condition (linear gingival erythema), appear to be unique to those with compromised immune systems [2-4].

Periodontal manifestations of the HIV+ infection were first described in 1987. Initially, the lesions receiving attention were HIV+ associated gingivitis (now known as Linear Gingival Erythema (LGE)) and HIV+ associated periodontitis (now known as Necrotizing Ulcerative Periodontitis (NUP)). The prevalence of NUP is low (<or=5%), and this lesion is associated with pronounced immunosuppression. The current focus on the periodontal manifestations of HIV+ infection centers on the rapid progression of chronic adult periodontitis in HIV+ patients [5-7], but attempts to identify the pathogenesis of this increased progression have not proven successful. For example, analysis of subgingival plaque for the presence of bacterial pathogens has failed to detect differences between HIV+ and HIV- patients. Recent evidence from Ochiai's (2009) study of the oral microbiota oral of HIV+ patients with periodontal disease found that periodontal diseases could act as a risk factor for HIV+ reactivation in latently infected individuals [8-10].

The prevalence of periodontal disease remains a question, with estimates of occurrence among HIV+ infected individuals ranging from 5% to 50%. It is not yet clear where in the spectrum of HIV+ disease these conditions occur, or which patients are at greatest risk for developing them. There is some evidence that NUP is associated with a low CD4 count (<200 cells/mm3) [11].

While dentists recommend that all people seek routine care to prevent oral health problems from developing, such care is particularly important for those living with HIV+. One rationale for this preventive measure is that individuals with a compromised immune system need to avoid bacterial infections. The two major oral health conditions, dental caries and periodontal disease, are both caused by bacteria and may be exacerbated by other factors. The most important components in the management of HIV+-associated gingival and periodontal disease should be the removal of local irritants from the root surfaces, debridement of necrotic tissues, and appropriate use of antibiotics [12,13].

Periodontal disease is also associated with hormonal changes as in pregnancy and diabetes. Few studies in the literature provide evidence of periodontal disease in pregnant women with HIV+ and its relation to pregnancy outcomes. Pregnant women with HIV+, because of their hormonal and immunosuppression status, are at increased risk for periodontal disease and poor pregnancy outcomes [14-17].

The purpose of this study was to evaluate the oral health status of pregnant women with HIV+ infections from Puerto that visit the CEMI (Center for Maternal Infant Studies or Centro de Estudios Materno Infantiles) obstetrics/gynecology (obgyn) clinic. Specific aims of the study were to: 1) evaluate the periodontal health status of pregnant women with HIV+ infections 2) evaluate the caries and caries risk status of pregnant women with HIV+ infections and 3) determine the dental treatment needs of this population of pregnant women with HIV+ infections.

## Methods

This cross-sectional pilot study was approved by the University of Puerto Rico Institutional Review Board (IRB). A convenience sample of 25 pregnant women with HIV+ infections was recruited at the CEMI obgyn clinic, (University of Puerto Rico) About 40 pregnant women with HIV+ infections visit this clinic each year. The present study recruited more than 50% of this population who visited the clinic during the year 2010. All subjects approached were provided with complete information about the study and they agreed to participate. An appointment was then scheduled with each subject for the initial dental examination and to fill out questionnaires. IRB and HIPAA (spell out both) consent forms were provided to all subjects and their Institutional Review Board and Health Insurance Portability and Accountability Act written consent was obtained. A dental examination was conducted by a calibrated dentist on all subjects to determine if caries were present, periodontal status and treatment needs. The caries exam was performed with a round explorer, dental mirror and a light source following Radike Scale for caries assessment. Following dental caries examinations, a periodontal examination was performed by a calibrated dentist of the gums of all subjects using a Florida probe. This periodontal examination obtained information on pocket depth, CEJ, bleeding on probing, plaque index and calculus index for all subjects Silness and Loe Indexes.

The definition for periodontal disease classification was the presence of 4 teeth with 1 site with bleeding on probing, pocket depth >4 mm and loss of attachment>4 mm (NIDCR criteria). After the dental examination, dental education was provided to each subject, their treatment needs were explained and an appointment in the CSOMI clinic was recommended to them in order to take care of their dental treatment needs. At the end of the study, a questionnaire about the CEMI clinic and the oral health quality of life was administered to all subjects. Statistical analysis was performed using the SPSS Program and an Excel data base. Descriptive statistics were used to present the caries and periodontal status. Correlation and chi square tests were used to study the association between CD4 and periodontal disease status at the probability level of 0.05.

## Results

81% of the women subjects had carious teeth with a mean DMFT of 12 and mean decay teeth (DT) of 4.8. Almost 40% of the women subjects also had retained roots, suggesting a high need for dental treatment (Table 1).

The prevalence of periodontal disease among women subjects was 72%, following the definition for periodontal disease classification: the presence of 4 teeth with 1 site with bleeding on probing, pocket depth >4 mm and loss of attachment>4 mm. One third of the women subjects showed a severe form of periodontitis. The mean number of teeth with pocket depth>4 mm was 6.95, the mean number of teeth with loss of attachment>4 mm was 7.66, and the mean sites of bleeding on probing was 12.09 (Table 2). In the subject sample, 3 women had more than 25% of periodontal sites that bled on probing (15% gingivitis), 7 women did not bleed on probing (33%), and 11 women presented gingival hyperplasia (52%). CD4 levels reported on the records of patients showed a statistically significant association with severity of periodontal disease (p<0.05).

## Discussion

This study demonstrated a high prevalence of periodontal disease among HIV+ infected, pregnant women in Puerto Rico, based on conservative criteria and the definition of periodontal disease. Together, these results demonstrate that pregnant women with HIV+ infections are in need of access to dental services, especially regarding their periodontal health.

Studies have shown that immunosuppression associated with HIV infection may increase an individual's risk for periodontal disease. Also, hormonal changes related to pregnancy increase the risk of periodontal inflammation. Although is still controversial and there is no consensus among researchers, the association between periodontal disease during pregnancy and increased risk of premature and low birth weight may also be a risk factor. Infections from periodontal disease and caries during pregnancy may provide an increased risk of localized infections and bacteremias that may pose extra risk on pregnancy outcomes.

Oral health is an important health issue during pregnancy. Thus, attention to the oral health needs of pregnant women with HIV+ infections should be encouraged in order to provide them with a greater quality of life, and better pregnancy progress and outcomes. Today, individuals and women living with HIV+ infections are living longer, and this increased life expectancy justifies a new vision for dental services and the prevention of dental disease in these patients. This study demonstrated the need for substantial dental treatment in this sample of pregnant women with HIV+ infections, as evidenced by the high a high caries rate (81%). and a large proportion of patients with retained roots (36%). Educational strategies to prevent and manage dental disease during pregnancy should be implemented during prenatal care.

## Conclusions

A high rate of non-treated dental disease was found in our sample of pregnant Puerto Rican women with HIV+ infections. Better efforts should be made to promote oral health strategies among such women during prenatal care.

## Figures and Tables

**Table 1:** Mean and percent distribution of caries indices In HIV+ PR pregnant women.

	HIV+ Pregnant PR Women
Mean DMFT	12%
Mean DT	4.8%
Mean MT	1.86%
Mean FT	5.3
Percentage of women with retained roots	36%
Percentage of women with missing teeth	63%
Percentage of women with carious teeth	81%

**Table 2:** Periodontal status of HIV+ PR pregnant women.

Periodontal variables	HIV+ pregnant women N=25
Generalized periodontitis	46.7%
Prevalence of periodontal disease	72%
Percentage of mild Periodontitis	33%
Percentage of moderate periodontitis	26%
Percentage of severe periodontitis	33%
Non periodontal disease prevalence	28%
Mean teeth with pocket depth>4mm	6.95
Mean teeth with loss of attachment>4 mm	7.66
Mean sites of bleeding on probing	12.09

## References

[R1] 1http://www.unaids.org/en/HIV_data/2011GlobalReport/2011-GR_es.asp

[2] Phiri R, Feller L, Blignaut E (2010). The severity, extent and recurrence of necrotizing periodontal disease in relation to HIV status and CD4+ T cell count. J Int Acad Periodontol.

[3] Ranganathan K, Magesh KT, Kumarasamy N, Solomon S, Viswanathan R (2007). Greater severity and extent of periodontal breakdown in 136 south Indian human immunodeficiency virus seropositive patients than in normal controls: a comparative study using community periodontal index of treatment needs. Indian J Dent Res.

[4] Lemos SS, Oliveira FA, Vencio EF (2010). Periodontal disease and oral hygiene benefits in HIV seropositive and AIDS patients. Med Oral Patol Oral Cir Bucal.

[5] Santo AE, Tagliaferro EP, Ambrosano GM, Meneghim MC, Pereira AC (2010). Dental status of Portuguese HIV+ patients and related variables: a multivariate analysis. Oral Dis.

[6] Vernon LT, Demko CA, Whalen CC, Lederman MM, Toossi Z (2009). Characterizing traditionally defined periodontal disease in HIV+ adults. Community Dent Oral Epidemiol.

[7] Sy AA, Freed BA, Chau FK, Marcus M (2011). National estimates of the characteristics of individuals infected with HIV who are likely to report and receive treatment for painful bleeding gums. Spec Care Dentist.

[8] Imai K, Victoriano AF, Ochiai K, Okamoto T (2012). Microbial Interaction of Periodontopathic Bacterium Porphyromonas gingivalis and HIV-Possible Causal Link of Periodontal Diseases to AIDS Progression. Curr HIV Res.

[9] Imai K, Ochiai K (20111). Role of histone modification on transcriptional regulation and HIV-1 gene expression: possible mechanisms of periodontal diseases in AIDS progression. J Oral Sci.

[10] Imai K, Ochiai K, Okamoto T (2009). Reactivation of latent HIV-1 infection by the periodontopathic bacterium Porphyromonas gingivalis involves histone modification. J Immunol.

[11] Mataftsi M, Skoura L, Sakellari D (2011). HIV infection and periodontal diseases: an overview of the post-HAART era. Oral Dis.

[12] González OA, Ebersole JL, Huang CB (2009). Oral infectious diseases: a potential risk factor for HIV virus recrudescence?. Oral Dis.

[13] Falasca K, Vecchiet F, Ucciferri C, Vignale F, Conti P (2008). Periodontitis and cytokine patterns in HIV positive patients. Eur J Med Res.

[14] Conde-Agudelo A, Villar J, Lindheimer M (2008). Maternal infection and risk of preeclampsia: systematic review and metaanalysis. Am J Obstet Gynecol.

[15] Ohlsson A (1989). Treatment of preterm premature rupture of membranes. A meta-analysis. Am J Obstet Gynecol.

[16] Vergnes JN (2008). Studies suggest an association between maternal periodontal disease and pre-eclampsia. Evid Based Dent.

[17] Martin R, Boyer P, Hammill H, Peavy H, Platzker A (1997). Incidence of premature birth and neonatal respiratory disease in infants of HIV positive mothers. The pediatric pulmonary and cardiovascular complications of vertically transmited Human Inmunodeficiency Infection study group. J Pediatr.

